# Benign hereditary chorea, not only chorea: a family case presentation

**DOI:** 10.1186/s40673-016-0041-7

**Published:** 2016-02-02

**Authors:** Jeanette Koht, Sven Olav Løstegaard, Iselin Wedding, Marie Vidailhet, Malek Louha, Chantal ME Tallaksen

**Affiliations:** Department of Neurology, Drammen Hospital, Vestre Viken Health Trust, Drammen, Norway; Institute of Clinical Medicine, University of Oslo, Oslo, Norway; Department of Neurology, Oslo University Hospital, Ullevål, Oslo, Norway; Department of Neurology, Salpêtrière Hospital, APHP, Sorbonne Universités, UPMC Univ Paris 6 UMR S 1127, Inserm U 1127, CNRS UMR 7225, Institut du Cerveau et de la Moelle épinière, Paris, France; Laboratoire de Biochimie et Génétique Moléculaire, Hôpital Armand Trousseau- AP-HP, Paris, France

**Keywords:** BHC, Benign hereditary chorea, Ataxia, Dystonia, Myoclonus, *NKX2-1* gene

## Abstract

**Background:**

Benign hereditary chorea is a rare disorder which is characterized by early onset, non-progressive choreic movement disturbance, with other hyperkinetic movements and unsteadiness also commonly seen. Hypothyroidism and lung disease are frequent additional features. The disorder is caused by mutations of the *NKX2-1* gene on chromosome 14.

**Case presentation:**

A Norwegian four-generation family with eight affected was identified. All family members had an early onset movement disorder, starting before one year of age with motor delay and chorea. Learning difficulties were commonly reported from early school years. The family presented with choreic movements at rest, but other movements were seen; myoclonus, dystonia, ataxia, stuttering and tics-like movements. All patients reported unsteadiness and ataxic gait was observed in two patients. Videos are provided in the supplementary material. Most affected family members had asthma and a subclinical or clinical hypothyroidism. Sequencing revealed a mutation in the *NKX2-1* gene in all eight affected family members.

**Conclusions:**

This is the first Norwegian family with benign hereditary chorea due to a mutation in the *NKX2-1* gene, c.671 T > G (p.Leu224Arg). This family demonstrates well the wide phenotype, including dystonia, myoclonus and ataxia.

**Electronic supplementary material:**

The online version of this article (doi:10.1186/s40673-016-0041-7) contains supplementary material, which is available to authorized users.

## Background

Benign hereditary chorea (BHC, MIM 118700) is an autosomal dominant movement disorder, characterized by early onset choreic movements and often hypotonia and delayed motor development [1–5]. In addition, other hyperkinetic movements may be present in up to 50 % of patients [5–10]. The hyperkinetic movements are typically aggravated by stress and disappear during sleep. BHC patients show minimal or no disease progression, and progressive dementia is not observed [11, 12]. Typically, an MRI of the brain shows no structural changes. The severity of symptoms varies widely, even in families with the same disease-causing mutation [5, 6, 13, 14]. ~Thirty percent of the patients appear to be sporadic in large cohorts due to reduced penetrance and de-novo deletions [5, 6].

The epidemiology of BHC is uncertain as the disease is rare and symptoms can be subtle. A prevalence estimate from Wales was 1 per 500 000, but this was done prior to genetic testing and the prevalence thus remains uncertain [4].

**Fig. 1:**
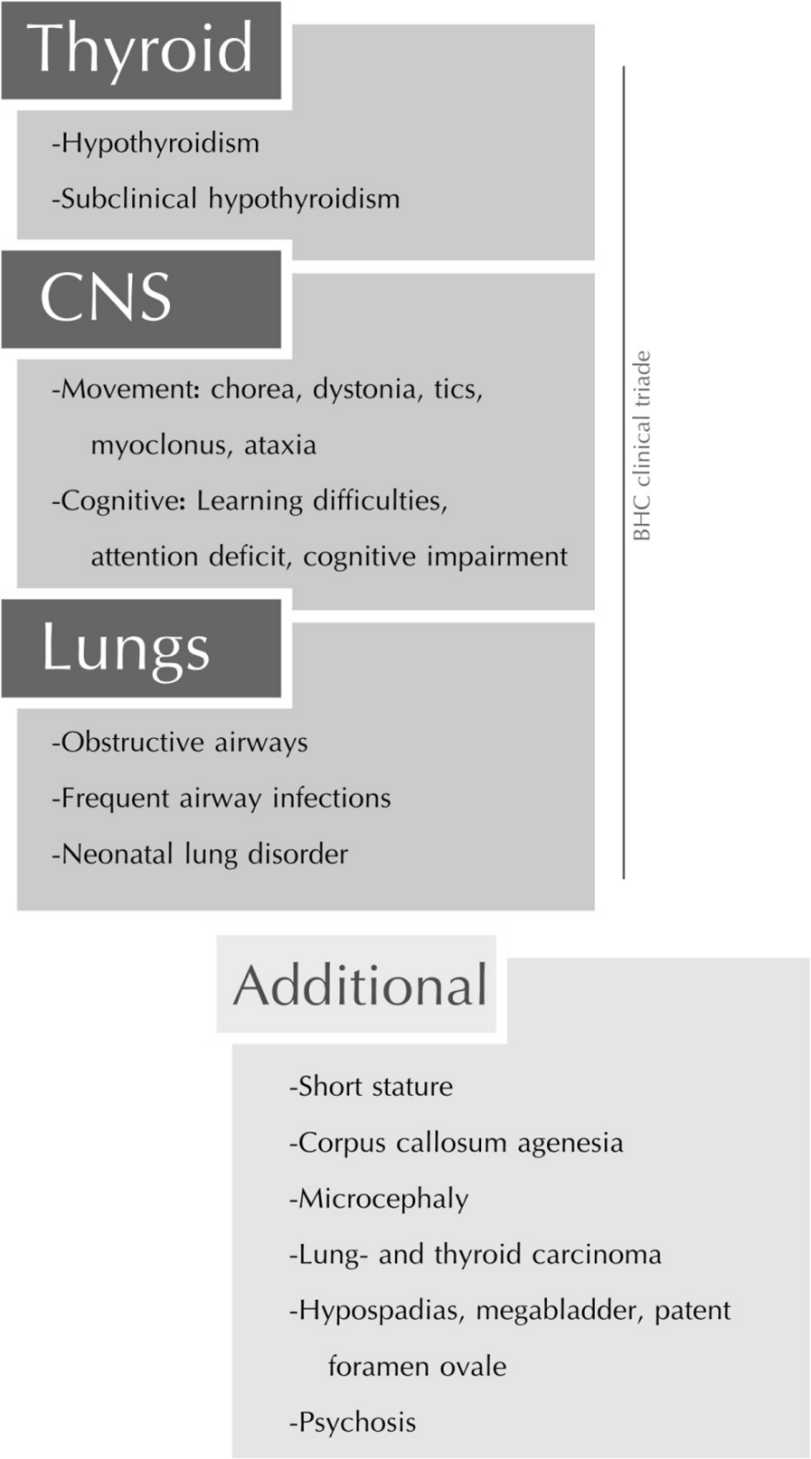
The clinical spectrum of Benign Hereditary Chorea

BHC is most often caused by mutations in the *NKX2-1* gene on chromosome 14 [15–17], but up to 50 % of the families initially given a clinical BHC diagnosis tested negative for mutations in the *NKX2-1* gene, however mutations in other genes may be associated with a similar phenotype, Table 1 [6, 7, 9, 18, 19]. As of today, sixty-one mutations have been described in the *NKX2-1* gene [6]. Genotype-phenotype correlations have not been found, but a milder phenotype is most often reported in patients with a missense mutation affecting the terminal regions of the protein compared to those with large deletions of the gene [1, 2, 5, 6, 13, 15–17, 20–22]. The gene encodes a homeodomain-containing transcription factor, TITF-1 (thyroid transcription factor-1), which is essential for the organogenesis of lung, thyroid and basal ganglia. The mutations are presumed to be disease causing due to haploinsufficiency [1, 2]. Mutations in the *NKX2-1* gene can also be associated with hypothyroidism and pulmonary disease as part of the “brain-lung-thyroid syndrome”[23]. The clinical spectrum in patients with mutations in the *NKX2-1* gene varies from the complete triad of brain-lung-thyroid syndrome (up to ~50 % of the patients), to brain and thyroid disease (~30 %), or isolated BHC (~20 %) which is the mildest expression of the syndrome [5], Fig. 1.

**Table 1:** Differential diagnoses for benign hereditary chorea

Diagnose^a^	Gene	Genetic clues^e^	Main clinical features
Benign hereditary chorea (BHC)	*NKX2-1*	AD, early onset	Hypotonia, chorea, lung and thyroid symptoms
Myoclonus dystonia (DYT11)	*SGCE*	AD, maternal imprinting	Myoclonus of short duration (<150 ms), dystonia
BHC like disorder	*ADCY5*	AD	Paroxysmal choreic/dystonic movements, facial myokymia
Huntington’s disease^b^	*HTT*	AD, anticipation	Chorea, athetosis, worsen over time, psychiatric symptoms and dementia
Huntington’s disease - like disorder 1–4^c^	*PRNP, JPH3, TBP*	AD/AR	Chorea, athetosis, worsen over time, psychiatric symptoms and dementia
Other Huntington's - like disorders	*RNF216*	AR	Cerebellar ataxia, behavioral problems, dementia, white matter lesions, hypogonadotropic hypogonadism, in some families chorea and athetosis
Ataxia telangiectasia	*ATM*	AR	Oculomotor apraxia, telangiectasia, dystonia
AOA1 (Ataxia with oculomotor apraxia 1)	*APTX*	AR	Early-onset cerebellar signs, sensory neuropathy, cognitive decline, and oculomotor deficits
Friedreich ataxia	*FXN*	AR	Sensory disturbances, spaticity, hyporeflexia, rare presentations with chorea and myoclonus
Hereditary ataxias (SCA1,2,3,6,7,17, DRPLA)	*ATXN1-3, CACNA1A,ATXN17, TBN, ATN1*	AD	Progressive ataxia, cerebellar (and brainstem) atrophy
Glucose transporter type 1 deficiency	*SLC2A1*	AD	Chorea and often mental retardation associated with a combination of paroxysmal ataxia, dystonia and/or epilepsy
Neurodegeneration with brain iron accumulation (NBIA)^d^	*PANK2*	AR/X-linked/AD	Typical MRI findings, dystonia, progression, cognitive decline

^a^There are many acquired conditions mimicking BHC in addition to this list

^b^In Huntington’s disease the juvenile forms often present with dystonia or parkinsonism

^c^Huntington disease like (HDL) 1–4; unknown gene in HDL3 (questioned entity), HDL4 the same as SCA17. HDL1 also known as inherited prion disease

^d^Mutations in ten genes can cause NBIA. Mutations in *PANK2* is the most common

^e^Many of these disorders appear sporadic due to reduced penetrance/age-dependent penetrance, variable expressivity and de-novo mutations

No curative treatment for this disorder is currently available, but there is one report of improvement in the hyperkinetic movements from tetrabenazine in a few patients who tried the medication [5]. In addition case articles have reported effect of levodopa [5, 20] and methylphenidate or haloperidol [24] in a few other subjects.

A large BHC cohort was described in 2012 [5], including this Norwegian family. The objective of this study is to provide a detailed description of the clinical data, medication response, neuropsychological features and videos from this first Norwegian BHC family.

### Case presentation

#### Methods and patients

**Fig. 2:**
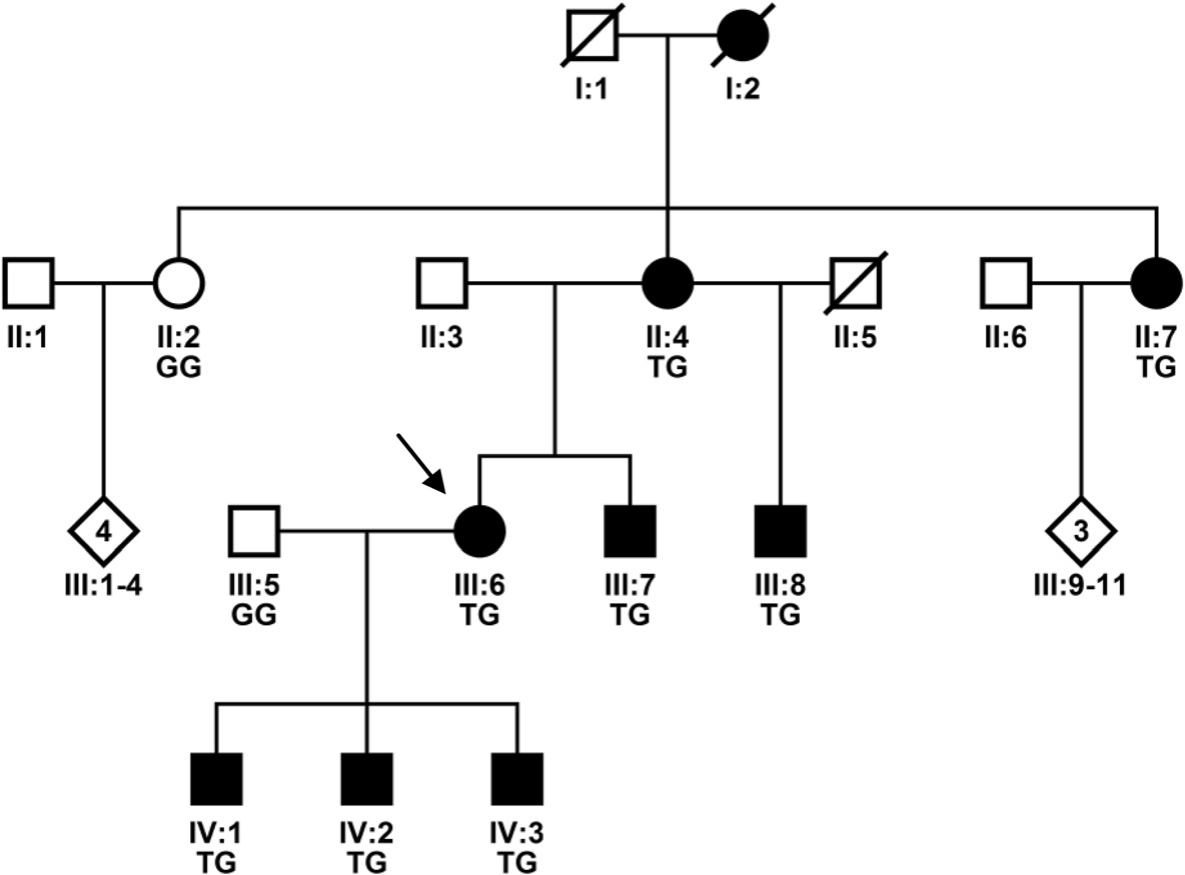
The pedigree of the family

During an ongoing study on movement disorders and hereditary ataxias in Norway [25], this four-generation family with eight affected subjects was referred to the department with possible hereditary ataxia, Fig. 2.

Videos were recorded from the examinations for all affected subjects and reviewed by experienced clinicians. The Scale for the Assessment and Rating of Ataxia (SARA-scale) was used to rate ataxia-related symptoms on a scale from 0 (no ataxia) to 40 (severe ataxia) [26].

Brain MRI scanning, mini-mental state test (MMS) and neuropsychological tests were performed in four available affected family members. Due to different ages, different test panels were used. WISC-IV (<18 years) and WASI (>18 years) were used for assessment of the Intelligence Quotient (IQ). Verbal Fluency Test, Stroop test and California Verbal Learning Test (CVLT) were performed to assess verbal executive function, executive functions with inhibition, selective attention, and verbal learning and memory. Paced Auditory Serial Addition Test (PASAT) was used to assess attention and working memory [27–29]. Due to geographical restrictions it was not possible to test all family members.

Blood samples were collected from all affected and unaffected family members. DNA was extracted from blood lymphocytes and sequenced using standard techniques. The sequence data have been described in an earlier publication [5].

Written informed consent was obtained from all included family members in accordance with the ethical agreement n° 129/04011 of the Regional Ethical Committee in Norway.

### Clinical data

Age of onset was during the first year of life in all subjects and hyperkinetic movements were the first reported symptoms in all. All affected subjects had delayed motor skills, but 4/8 reached the walking milestone within normal range. The movement disorder was reported to be stable, but observed to be more prominent in the youngest generation. All patients reported increased symptoms with stress and two reported some relief in hyperkinetic movements with ethanol consumption. Learning difficulties in early school age was reported in 7/8. Only one of the patients had an academic education. Clinical data are given in Table 2.

**Table 2:** Clinical data of the affected family members

	SARA score	Hyperkinetic movements^a^	Thyroidea	Lungs	MMS	IQ	Verbal IQ (percentile)	Performance IQ (percentile)
II:4	12.5	Dystonia	Normal values, treated earlier	Asthma	NA	NA		
II:7	10.5	Dystonia, mild ataxia	Hypothyreoses, treated	Asthma	NA	NA		
III:6	6	Myoclonus, mild ataxia	Compensated values^b^	Asthma	30/30	99	41th	59th
III:7	7	Dystonia	Compensated values^b^	Frequent infections	NA	NA		
III:8	5	Dystonia, stuttering, tics,	Normal values	No lung problems	NA	NA		
IV:1	5.5		Normal values	Asthma	0/30	90	14th	95th
IV:2	5		Normal values	Asthma	30/30	81	NA	NA
IV:3	7		Compensated values^b^	Asthma	30/30	86	30th	68th

^a^In addition to chorea

^b^Normal fT4, but elevated TSH

**Fig. 3:**
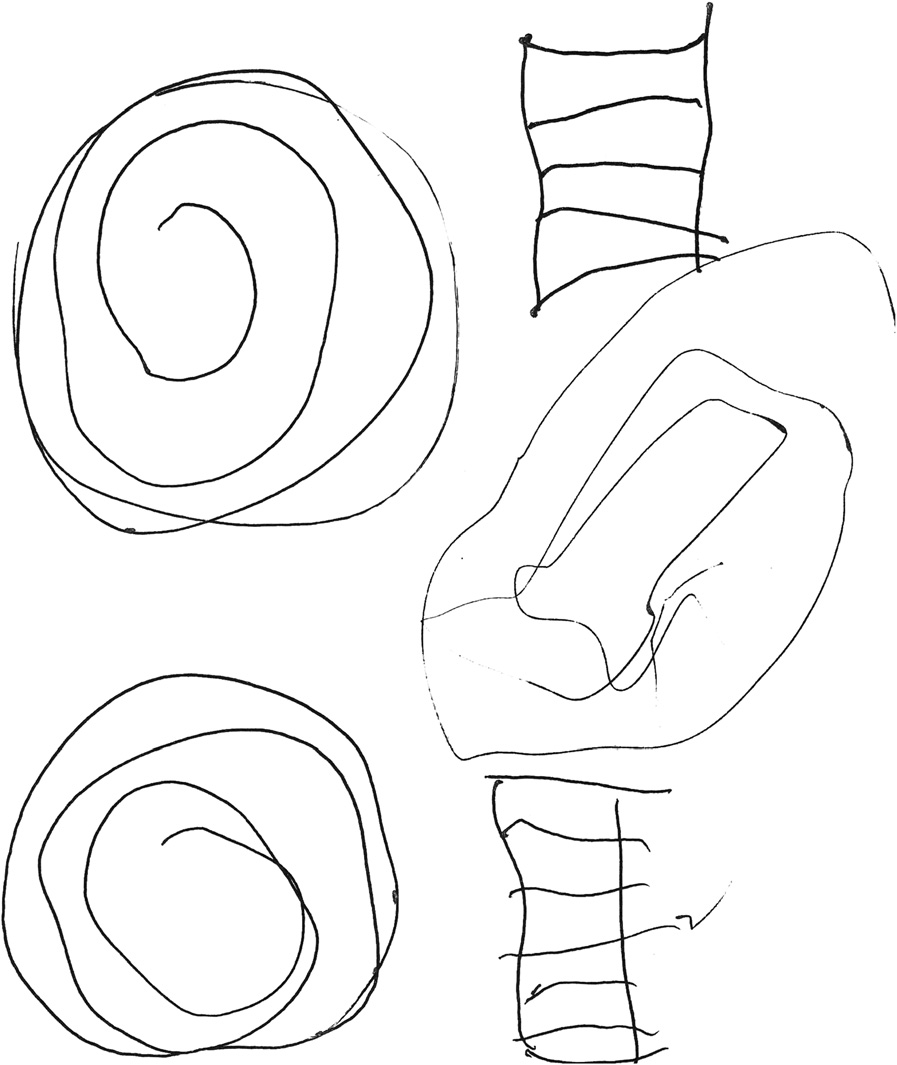
A typical Archimedes spiral drawing by one of the affected subjects

The mean age at the time of examination was 36 years with an average of 33.5 years (range 7–63 years). All subjects had involuntary movements with chorea at the time of examination. Chorea was the most prominent hyperkinetic movement in the three youngest, Additional file 1: Video 1. The older generations exhibited myoclonus (one subject), a mixture of dystonia and chorea (three subjects), Additional file 2: Video 2, and tics, stuttering and dystonia (one subject). All subjects were unsteady and unable to walk ten steps in tandem and two subjects had mild gait ataxia, Additional file 3: Video 3. Saccadic pursuit was consistently observed in all when tested for slow pursuit. Hanging reflexes or increased reflexes were seen in 7/8, Additional file 2: Video 2. Archimedes spiral drawing was inaccurate in all the affected subjects, illustrated by subject IV-2, Fig. 3.

No changes in symptoms were observed in the index patient and the youngest generation at two years follows up time.

Brain MRI’s were normal in the four scanned subjects.

No short stature, microcephaly, epilepsy, pulmonary carcinoma or psychiatric disorders were reported or observed in the family.

Levodopa failed to improve the involuntary movements in II:7 and III:6 (1.5-3 mg/kg/day). Clonazepam (0.5 mg/day) relieved the hyperkinetic symptoms in III:7 to some degree, but III:6 observed only side-effects (dizziness and fatigue). Tetrabenazine was tested in increasing doses in IV:2 and IV:3 (up to 0.35 mg/kg/day). Side-effects appeared after a few days and the medication was discontinued after one week with nocturnal bed-wetting for IV:2 and depressive symptoms in IV:3. Topiramate was tried in two subjects with BMI > 30. They reported some reduction in involuntary movements, but Topiramate was discontinued after one month due to side effects with depressive symptoms and fatigue in both.

#### Neuropsychological testing

Neuropsychological test results for four subjects are shown in Table 2. Verbal IQ was consistently lower than performance IQ in all. The index subject showed reduced working memory and attention in the PASAT test, scoring 29/60.

Subjects IV:1, IV:2 and IV:3 were tested with WISC-IV and all showed working memory and processing speed in the lower range. These three subjects performed in the lower normal range in verbal memory and verbal fluency, while visual memory and understanding was in the middle normal range.

All four reported concentration problems, as reflected in reduced attention and concentration. The most significant attention impairment was seen in patient IV:1, with Stroop results corresponding to the 2^nd^ percentile.

#### Case description

The index patient was referred to our clinic due to unsteadiness and clumsiness from early school age. She wanted a second opinion when she was 34 years old on her “hereditary ataxia” diagnosis.

The hyperkinetic movements started in infancy and her parents described her as a restless baby. When she was examined, at age 34 and at follow-up at age 36, she had obvious hyperkinetic movements, most prominent in the trunk. She reported no obvious progression, but worsening in the involuntary movements during adolescence and pregnancy. She described her movements as a lack of ability to keep her body at rest. She experienced her first pneumonia at the age of 34 years and was diagnosed with asthma at the age of 36 years and for which she received medications.

The most noticeable findings at the first examination were myoclonic movements in the trunk, unsteadiness with abnormal gait and the inability to walk ten consecutive steps in tandem. She was, however, able to stand with her feet together. She had intermittent sway when sitting and subtle choreic movements were observed in her hands. Her SARA score was 6/40 points, with unsteadiness and very mild ataxia in extremities. Her speech was normal.

After two years follow-up the examination was similar with the same SARA score (6/40 points).

Elevated levels of thyroid-stimulating hormone in serum (TSH), with normal thyroxin (free T_4_) values were measured.

#### Genetics

All affected subjects carried a point mutation in exon 3, c.671 T > G, p.Leu224Arg, previously reported for this family [5]. Array CGH was normal in the index patient.

## Conclusions

This family presented a broad spectrum of hyperkinetic movements with chorea as the most prominent symptom in the youngest generation, but with combinations of dystonia, myoclonus, mild ataxia and stuttering in the older generations. At the time of examination, all affected subjects exhibited unsteadiness, and were unable to walk in consecutive tandem steps. The affected index family member was originally diagnosed with hereditary ataxia, due to family history and mild ataxia. The predominant feature, chorea, was probably overseen in this family and delayed the identification of the correct etiology. In addition neither progression nor atrophy of the cerebellum on MRI scans were observed in this family. The diagnosis was therefore revised. This emphasizes the importance of accurate phenomenology and the importance of examination of more family members due to intrafamiliar clinical heterogeneity, which is commonly seen in monogenetic disorders, Table 1 [30].

Even though myoclonus, dystonia or chorea have been reported in various genetic forms of hereditary ataxia, mutation screening of the *NKX2-1* gene (BHC) should be made in cases of juvenile “ataxia” with no other cause found. There is a wide phenotypic heterogeneity in BHC families, in addition to frequent de novo mutations.

Conversely, BHC may share some clinical features with other hyperkinetic disorders, especially where a mixture of choreic movements, myoclonus and dystonia is concerned, Table 1 [6, 9, 11, 18, 31]. An alternative diagnosis in this family may have been myoclonic dystonia (DYT11), as myoclonus and dystonia were additional movements in generation II and III. The lack of maternal imprinting in the pedigree and typical choreic movements in the youngest generation made however the diagnosis unlikely [8].

In this family a diagnosis of hereditary ataxia was less likely as the hyperkinetic feature chorea was the most prominent symptom and finding, even though all subjects were unsteady and two had mild ataxia. Hyperkinetic movements, autosomal dominant inheritance pattern, early onset, normal MRI scans and minimal progression made us therefore consider BHC [6]. A correct diagnosis is of great importance for the patients, especially as many differential diagnoses, including early onset ataxia, often have a more severe prognosis compared to BHC [30]. However, slight progression in children could be masked by overall improved motor skills and can therefore be difficult to assess.

The opposite may also be true as there are some families with BHC phenotypes that do not have *NKX2-1* mutations. Some families have been reported to have *ADCY5* mutations [18, 19]. The main distinctive features that may help to differentiate the *ADCY5* families from *NKX2-1* families are facial myokimias, paroxysmal movement disorders, spasticity, more prominent dystonic features and clinical progression in the *ADCY5* families reported.

All affected members of the family reported concentration problems and the neuropsychological domains attention, working memory and processing speed were indeed affected. The patients who were extensively cognitively tested were consistently better in executive and visuospatial functions as compared to verbal functions, with verbal IQ being consistently lower than executive performance IQ. Although previous reports on cognition in BHC have been conflicting, it has still not been established whether the impairment is a direct consequence of the different mutations, whether it is biased by the motor symptoms or as a consequence of hypothyroidism in some of the subjects. A drawback in this and previous reports is the low number of patients available for neuropsychological testing.

Even though a broad specter of cognitive profiles are reported in previous papers on BHC patients, the phenotype usually shows a mild cognitive decline and no alteration over time has been reported. Normal cognitive status is reported to be more likely in BHC compared to other chorea genotypes, and is considered to be a good clue to differentiate BHC from neurodegenerative forms of chorea [31–33]. Our observations suggest that, even when patients have some alterations in cognitive tests, they have completely different cognitive profiles compared to the hereditary neurodegenerative forms of chorea [6].

To date, the most successful treatments reported in a few individual patients with BHC are tetrabenazine and levodopa [5, 20, 24]. These medications were tested in some of the patients, but only side-effects and no or little benefit on the hyperkinetic movements was observed. Therefore no conclusions or guidelines can be drawn from this family or previous reports.

The best treatment options for most patients with BHC are probably still physical activity, physiotherapy, multidisciplinary facilitation at school and at work, and yearly lung and thyroid controls and treatment if needed.

In conclusion, although the neurological spectrum of BHC is more and more delineated, our family illustrates that hyperkinetic movement in addition to chorea can be prominent in these families. Therefore, we suggest considering BHC with mutations in the *NKX2-1* gene in the diagnostic workup also in those patients with hyperkinetic movements such as early onset dystonia and ataxia.

## Consent

Written informed consent was obtained from patients for publication of this Case report. A copy of the written consent is available for review by the Editor-in-Chief of this journal.

## Additional files

Additional file 1: Video 1. Video 1 illustrates choreic movements in one of the patients. (MOV 6721 kb) Additional file 2: Video 2. Video 2 illustrates chorea, dystonia and hanging patellar reflex in one of the patients. (MOV 9599 kb) Additional file 3: Video 3. Video 3 illustrated mild ataxia when performing the heel–knee–shin test. (WMV 3646 kb)

## Abbreviations

BHC: Benign hereditary chorea
